# Exciting Times: New Advances Towards Understanding the Regulation and Roles of Kainate Receptors

**DOI:** 10.1007/s11064-017-2450-2

**Published:** 2017-12-21

**Authors:** Ashley J. Evans, Sonam Gurung, Jeremy M. Henley, Yasuko Nakamura, Kevin A. Wilkinson

**Affiliations:** School of Biochemistry, Centre for Synaptic Plasticity, Biomedical Sciences Building, University of Bristol, Bristol, BS8 1TD UK

**Keywords:** Kainate receptors, GluK2, Trafficking, RUSH, Synaptic transmission, Synaptic plasticity

## Abstract

Kainate receptors (KARs) are glutamate-gated ion channels that play fundamental roles in regulating neuronal excitability and network function in the brain. After being cloned in the 1990s, important progress has been made in understanding the mechanisms controlling the molecular and cellular properties of KARs, and the nature and extent of their regulation of wider neuronal activity. However, there have been significant recent advances towards understanding KAR trafficking through the secretory pathway, their precise synaptic positioning, and their roles in synaptic plasticity and disease. Here we provide an overview highlighting these new findings about the mechanisms controlling KARs and how KARs, in turn, regulate other proteins and pathways to influence synaptic function.

## Introduction

Glutamate is the major excitatory neurotransmitter in the CNS and participates in nearly all aspects of brain function. There are three major subclasses of ionotropic glutamate receptors, kainate receptors (KARs), AMPA receptors and NMDA receptors. KARs are widely distributed throughout the brain and, depending on the cell type in question, they can be localised at pre-, post- and/or extrasynaptic sites. In general, presynaptic KARs modulate both excitatory and inhibitory neurotransmitter release, postsynaptic KARs contribute to excitatory neurotransmission and extrasynaptic KARs play a role in determining neuronal excitability (for reviews of KAR physiology see [1–4]). For reasons that remain unclear, compared to AMPARs and NMDARs, synaptic KAR ionotropic responses are highly restricted to subsets of excitatory synapses. For example, some neurons, including CA1 pyramidal neurons and dispersed hippocampal cultures display robust KAR currents following kainate application [5, 6] but they lack synaptically-evoked ionotropic postsynaptic excitatory post-synaptic currents KAR_(EPSCs)_ [6–8]. Intriguingly, it has been proposed that KAR_(EPSCs)_ are present only at synapses that do not contain AMPAR_(EPSCs)_ [9, 10].

Remarkably, despite their classical ion channel structure, KARs can also signal via a non-canonical G-protein coupled metabotropic cascade [11]. In contrast, postsynaptic metabotropic KAR signalling is much more widespread than ionotropic KAR signalling [12, 13] and recent discoveries have highlighted their previously unsuspected roles in neuromodulation. For example, they regulate inhibitory transmission by controlling surface expression of the chloride transporter KCC2 [14] and mediate certain forms of synaptic plasticity [15, 16]. For extensive reviews of metabotropic KARs see [4, 17].

Our aim here is to provide an overview of recent advances in understanding the cellular regulation of KARs and their interacting proteins, and how these processes influence KAR-mediated synaptic transmission and plasticity. We highlight what is known, what remains to be established, and outline the future perspectives for KAR research and how it will impact on our understanding of brain function and dysfunction in disease.

## Nomenclature of KAR Subunits

Since their initial cloning [18, 19] the names of specific KAR subunits have changed to conform to a simpler, more systematic naming system. This can lead to considerable confusion in the field since many seminal early papers use the old nomenclature. Nonetheless, since 2009, the International Union of Basic and Clinical Pharmacology (IUPHAR) naming system has been almost universally adopted. Briefly, the subunits formerly most commonly referred to as GluR5, GluR6, GluR7, KA1 and KA2 are now named GluK1, GluK2, GluK3, GluK4 and GluK5, respectively, and the genes encoding these proteins are named *GRIK1-5* [20].

## KARs from Birth to Maturity

### KAR Structure and Assembly

KARs are heteromeric assemblies containing four subunits. Each subunit has a large extracellular N-terminal domain (NTD), helical transmembrane domains (TMD) including three membrane spanning domains (M1, M3 and M4) and a membrane re-entrant domain (M2), and an intracellular C-terminal domain (CTD). The latter part of the NTD (the last ~ 150 amino acids, S1) together with the extracellular loops between M3 and M4 (S2) form the ligand-binding domain (LBD) [21]. Recently, detailed structural information has been gained by solving the crystal structure of kainate receptor subunits and this is reviewed in detail elsewhere [22–25].

#### Heteromeric Assembly

Following protein synthesis, the NTDs initiate receptor assembly in the endoplasmic reticulum (ER) by facilitating dimer formation, and the dimerization of two dimers then leads to the formation of tetrameric receptors. Based on affinity for their ligand, KAR subunits have been grouped into low affinity (GluK1-3) and high affinity (GluK4-5) receptors. Studies on recombinant systems have shown that low affinity GluK1-3 subunits can form ion channels as both homomers and heteromers but high affinity GluK4 and GluK5 subunits can only form heteromeric functional ion channels when complexed with the low-affinity subunits [19, 26, 27]. The most abundant subunit combination in the brain comprises GluK2 and GluK5. This occurs, at least in part, because widely distributed contacts within the NTD of GluK2 and GluK5 favour the assembly of functional heteromeric receptors over homomeric receptors [28].

#### Alternative Splicing

Regions within the N- and C- terminal domains of KAR subunits can undergo alternative RNA splicing. For instance, the extracellular N-terminal domain of GluK1 can produce two variants, GluK1_1_ and GluK1_2_ [29], while the C-terminus has four splice variants, GluK1a, GluK1b, GluK1c and GluK1d [30, 31]. Splice variants have also been reported at the C-termini of both GluK2 and GluK3; GluK2a/GluK2b/GluK2c and GluK3a/GluK3b, respectively [32–34]. C-terminal alternative splicing of KAR subunits has been shown to greatly affect the ability of receptors to exit the ER and accumulate at the cell surface. Furthermore, different C-termini facilitate distinct protein–protein interactions and it is likely they provide mechanisms for nuanced tuning of specific KARs at particular locations [35–37].

#### RNA Editing

In addition to splicing, further diversity arises from varying degrees of RNA editing in GluK1 and GluK2 subunits [38, 39]. For example, Q/R editing in the pore-lining region of GluK2 results in a change from the genomically encoded glutamine residue to an arginine. This change alters the properties of the resultant KAR from calcium permeable to calcium impermeable and also alters the biophysical properties of the channel [40]. Furthermore, GluK2 Q/R editing reduces its ability to assemble with other subunits, leading to its accumulation as monomers and dimers that are retained in the ER [41].

It is well established that GluK1/GluK2 editing is developmentally controlled through regulation of the enzyme that also catalyses GluK1/2 RNA editing, ADAR2 [42–44]. For an excellent recent review see [45]. ADAR2 levels are low in embryonic brain and during development ADAR2 levels increase [46]. After birth ~ 80% of GluK2 and 40% GluK1 are edited, which leads to fewer surface KARs and lower conductance and Ca^2+^ permeability [47]. Furthermore, recent evidence has suggested that the ADAR2 dependent Q/R editing of GluK2 is also dynamically regulated during homeostatic scaling [48]. Suppression of synaptic activity with TTX results in upscaling of KAR surface expression, which is, at least in part, due to reduced Q/R editing of GluK2 [48]. Therefore, this developmental and homeostatic regulation of GluK2 Q/R editing likely control processes such as synaptogenesis [49, 50], plasticity [40] and pathology [47].

### KAR Trafficking Through the Secretory Pathway

The accurate and timely delivery of KARs to specific pre-, post- and extrasynaptic locations is fundamental to many aspects of neuronal function. Most research efforts have focused on the processes of transcription, endocytosis, recycling and degradation (for reviews see [4, 51]). However, after assembly, tetrameric KARs need to traffic through the secretory pathway to reach the cell surface and be appropriately targeted. Until recently, it was unknown whether these early KAR trafficking steps occur locally in dendrites and, importantly, how these processes are regulated.

#### Local Dendritic Translation and Secretory Pathway Trafficking

In neurons, mRNAs can be trafficked to distant sites in axons and dendrites for local translation and processing [52–57]. A range of neuronal transmembrane proteins, including AMPARs, NMDARs and GABA_B_Rs, can be translated using both somatic and dendritically localised ribosome patterned rough ER. They then traffic from the ER using dendritic ER exit sites and utilise the somatic Golgi or dendritic Golgi outposts for mature glycosylation [48, 58–62]. Importantly, all of the secretory pathway machinery appears to be present in neurites as iGluRs can mature in isolated dendrites [63]. For example, AMPAR mRNAs traffic into dendrites, under the control of synaptic activity, to create local utilizable pools of mRNA for local translation [64].

**Fig. 1:**
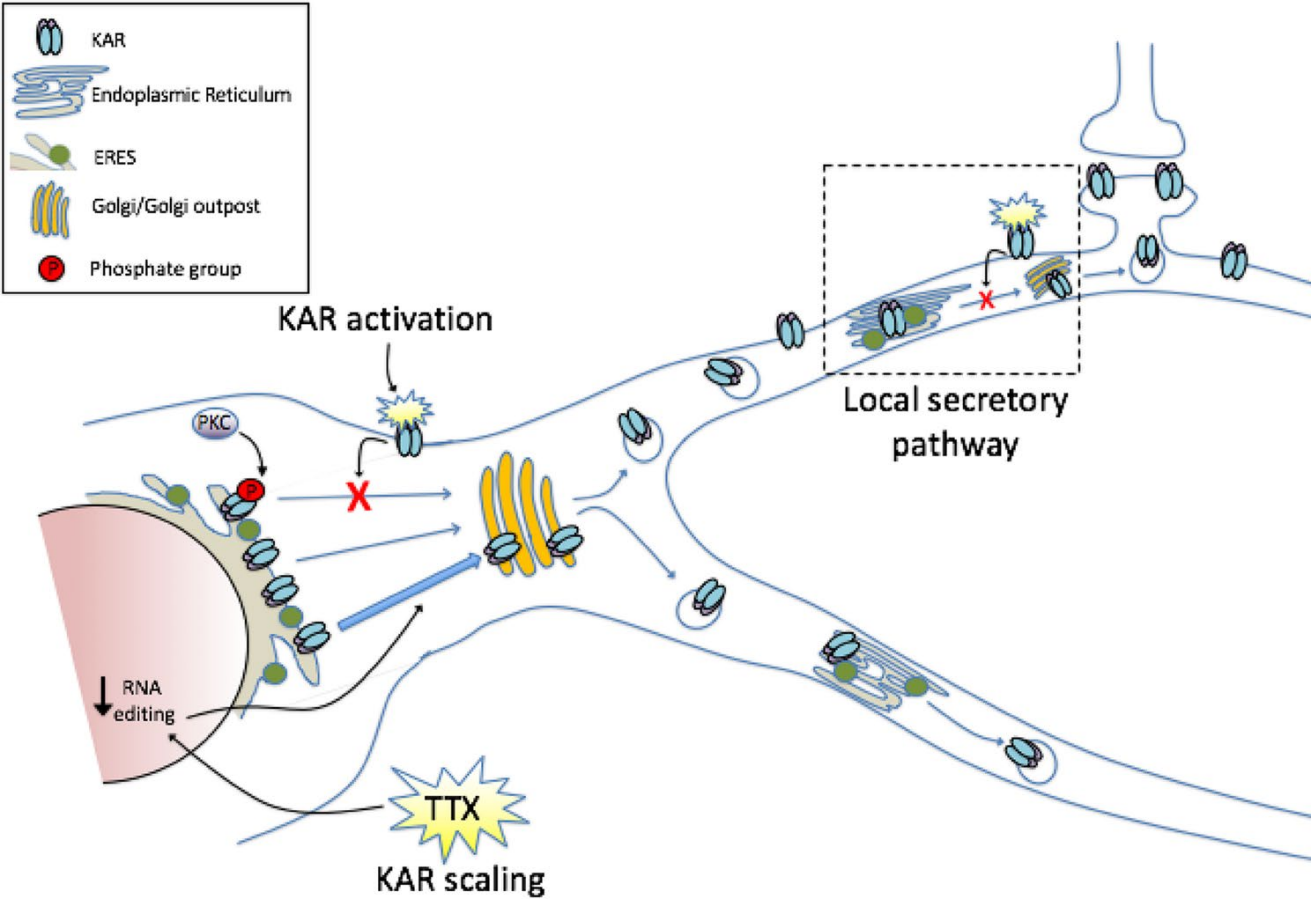
Control of KAR trafficking through the secretory pathway. KAR trafficking is controlled at multiple levels under basal and activity-dependent conditions. PKC phosphorylation of the GluK2 subunit at S846 and S868 reduces ER exit of GluK2-containing receptors. Activation of surface KARs leads to a feedback mechanism that reduces forward trafficking of KARs from the ER, via a mechanism that requires the PDZ ligand of GluK2. Furthermore, induction of synaptic scaling via inhibiting neuronal activity with TTX leads to upscaling of KARs, at least in part via reduced RNA editing of the GluK2 subunit, favouring forward trafficking of GluK2-containing KARs. Finally, in addition to using somatic Golgi for post-ER processing, KARs can also use local secretory pathway systems in dendrites

Recently, using the RUSH system, which allows the synchronous release and visualization of cargo proteins trafficking through the secretory pathway [65, 66], it has been demonstrated that GluK2-containing KARs utilise these local secretory pathway systems for their delivery to the cell surface [48] (Fig. 1). However, the functional consequences that result from KARs utilising these local secretory pathway systems remain to be established.

#### Activity Dependent Secretory Pathway Trafficking

Although there is a strong base of knowledge about the activity-dependent regulation of KAR endocytosis and recycling [13, 15, 67–71], compared to AMPARs [72] and tsVSVG cargo [73], little is known about the activity-dependence of secretory pathway trafficking of KARs. A very recent study reported that secretory pathway KAR trafficking is indeed highly regulated in multiple different cellular activity contexts [48]. Activation of surface KARs results in a decrease in secretory pathway trafficking of de novo GluK2-containing KARs from the ER to the cell surface, demonstrating that KAR secretory pathway trafficking is subject to a negative feedback mechanism controlling KAR surface levels. Mechanistically, this pathway is dependent on the PDZ ligand of GluK2, since a mutant lacking this protein interaction site is insensitive to the effects of kainate on secretory pathway trafficking, however the GluK2 interacting partner responsible remains to be determined [48].

#### Roles of GluK5 in Trafficking

As well as interactions with the GluK2 subunit, interactions with GluK5 also determine flux through the secretory pathway. ER retention sequences in the C-terminus of GluK5 facilitate its interaction with the COPI coat complex, driving retrograde Golgi to ER trafficking of KARs, and acting as an ER retrieval mechanism [74]. This interaction is disrupted by the heteromerization of GluK2 with GluK5 and binding to 14-3-3ζ, which promotes forward trafficking to the cell surface, both driving ER exit and favouring assembly of heteromeric KARs [74].

#### Post-translational Modifications

Further tight control of the ER exit and secretory pathway trafficking of KARs is also provided by post-translational modifications, most notably PKC phosphorylation of GluK2 at residues S846 and S868 [48, 75], which act to restrict forward trafficking of GluK2-containing KARs.

Thus, far from being a passive process, ER exit of KARs and flux through the secretory pathway is emerging as a major point of regulation in determining the surface expression of KARs under basal and activity-dependent conditions (summarised in Fig. 1).

## Synaptic Positioning and Function of KARs

**Fig. 2:**
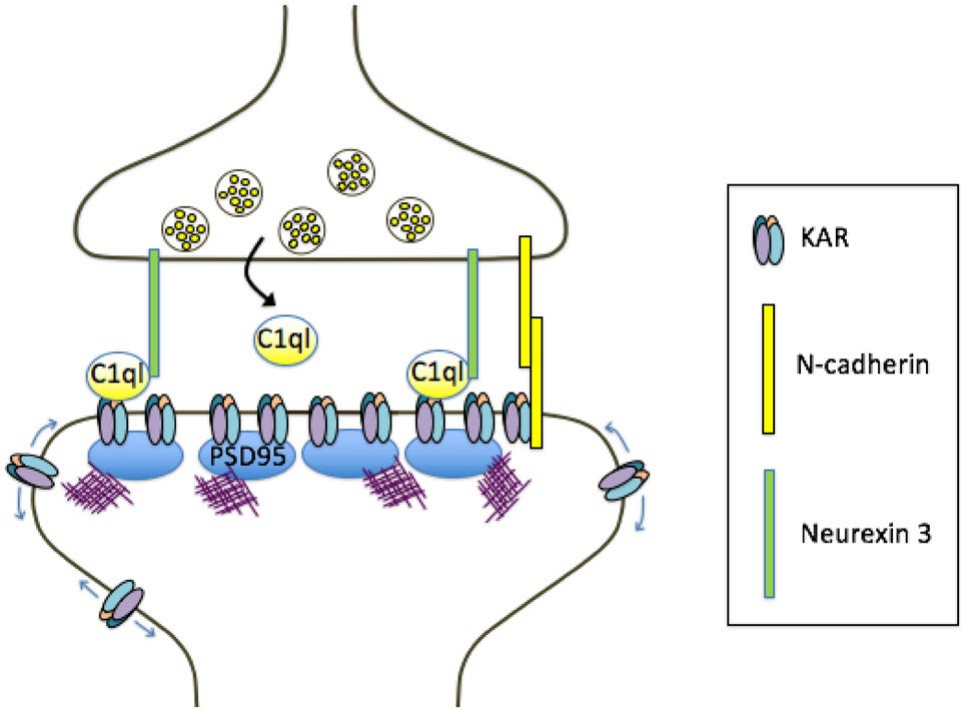
Mechanisms of KAR synaptic localisation. At mossy fibre-CA3 (MF-CA3) synapses, presynaptically released C1ql proteins cluster KARs through binding to KAR subunit extracellular domains, and forming a tripartite trans-synaptic complex with presynaptic neurexin 3. KAR synaptic incorporation is also promoted through interactions between the GluK2 C-terminus and the trans-synaptic adhesion molecule N-cadherin, and through interactions between the C-termini of GluK1, GluK2 and GluK5 (and potentially Neto1) and PSD95

Given the highly-ordered targeting and localisation of KARs at distinct synaptic and sub-synaptic compartments, a key question in the field relates to how KARs are targeted to, and retained at, specific pre- or postsynaptic locations. While many of the factors that mediate this distribution are yet to be determined, as discussed below, recent data suggest that structural aspects of the receptor subunits, the presence of Neto auxiliary proteins in the KAR complex and the secreted C1q-like proteins play important roles in these processes (Fig. 2).

### Postsynaptic KARs

#### Role of the GluK2 C-Terminus in Synaptic Localisation

In the cerebellum, KARs are located post-synaptically on cerebellar granule cells, which receive inputs from mossy fibres. These receptors comprise GluK2/5 and the auxiliary subunit Neto2 [76] (see below). Synaptic localisation of this complex is dependent on the GluK2 subunit, since ablation of GluK5 or Neto2 has no effect on levels of GluK2/Neto2 or GluK2/GluK5, respectively, in the postsynaptic density (PSD) fraction [76, 77]. Furthermore, knock-in mice in which the intracellular C-terminus of GluK2 is replaced with the C-terminus of the AMPAR subunit GluA1 do not exhibit synaptic KAR responses in cerebellar slices [77]. Notably, whole cell KAR currents were unaffected, demonstrating a specific role for the GluK2 C-terminus in synaptic incorporation of KARs at cerebellar synapses but not in receptor surface expression [77]. Furthermore, these mice show reduced postsynaptic KAR responses at MF-CA3 synapses in the hippocampus, the prototypical KAR-containing synapse in the brain, but no reduction in total KAR responses, again indicating a specific role for the GluK2 C-terminus in synaptic incorporation, but not surface expression, of KARs [77].

#### Roles of GluK4 and GluK5 in the Synaptic Specificity of KARs

While the C-terminus of GluK2 seems to be necessary for synaptic incorporation, synapse specificity in the hippocampus has been shown to be dependent on the GluK4 and GluK5 subunits. Postsynaptic KARs at MF-CA3 synapses are lost in mice lacking both of these subunits [78]. Moreover, in GluK4/5^−/−^ mice there is a redistribution of GluK2 immunolabelling to more distal dendrites in CA3 pyramidal neurons [77], suggesting GluK4/5 are crucial determinants of the synapse specificity of KARs in the hippocampus. Interestingly, viral re-expression of GluK5 in the hippocampus of GluK4/5^−/−^ mice rescued GluK2 signal in stratum lucidum, but this did not occur for a GluK5 chimera in which the extracellular N-terminus was replaced with that of GluK2 [77], which the authors attribute to the ability of the N-terminus of GluK5 to bind to the mossy fibre-enriched C1q-like proteins (see below).

### Proteins that Interact with KARs to Define Postsynaptic Localisation

#### N-Cadherin

The fact that MF-CA3 synapses in GluK2^−/−^ mice lack postsynaptic KAR responses [79] has allowed examination of the factors required for their effective synaptic positioning by re-expressing wild-type or mutant GluK2 in CA3 neurons. The last 20 amino acids of GluK2 are required for KAR incorporation at MF-CA3 synapses [80] and this region mediates interactions between GluK2 and the neuronal cell adhesion molecule N-cadherin [81]. At MF-CA3 synapses in wild-type mice, expression of a dominant-negative N-cadherin reduced KAR EPSCs, as did knockdown of N-cadherin mediated by expression of Cre recombinase in CA3 cells from N-cadherin floxed mice [80]. Together, these findings demonstrate GluK2 binding to N-cadherin is a key determinant of GluK2 recruitment to MF-CA3 synapses.

#### PSD95

The synaptic scaffold protein PSD95 binds to the C-terminal PDZ ligands of the core KAR subunits GluK1, GluK2 and GluK5 [82, 83]. PSD95 also binds to the auxiliary subunit Neto1 [84], and accelerates the recovery of GluK2 from desensitisation [85]. The KAR component of the EPSC at MF-CA3 synapses is reduced in PSD95 knockout mice, suggesting a role for this interaction in KAR synaptic localisation [86]. However, whether this is due to direct binding of KARs to PSD95 and, if it is, which subunits are responsible, remains to be determined. Moreover, as discussed below, regulation of the interaction between GluK5 and PSD95 is required for long-term depression (LTD) of KARs at MF-CA3 synapses [87].

#### C1ql

The C1q-like proteins are a family of secreted synaptic organisers [88]. Two members of this family, C1ql2 and C1ql3, are highly expressed by hippocampal mossy fibres and contribute to the localisation of KARs to mossy fibre synapses in CA3 neurons [77, 89]. In heterologous cells, C1ql2 and 3 bind to the N-terminal domains of GluK2 and GluK4 [89], although another study instead observed binding to GluK5 [77]. C1ql2 and 3 are present at MF-CA3 synapses, and their levels are reduced in slices from GluK2^−/−^ or GluK4^−/−^ mice, suggesting GluK2 and GluK4 act as binding sites for C1ql2/3 [89]. Moreover, GluK2/3 immunoreactivity is markedly decreased in CA3 stratum lucidum in C1ql2/3 knockout mice, and KAR EPSCs at MF-CA3 synapses are reduced to levels similar to those observed in GluK2^−/−^ mice. The presynaptic adhesion molecule neurexin 3 binds to secreted C1ql2/3 and a neurexin 3-C1ql-KAR complex was isolated in co-cultures of HEK293 cells expressing neurexin 3 and C1ql2/3 and neurons [89]. Thus, specific secretion of C1ql proteins at mossy fibres provides the basis for a trans-synaptic complex that clusters KARs postsynaptically at MF-CA3 synapses (Fig. 2).

#### Neto1 and Neto2

Neto1 and Neto2 are single pass transmembrane proteins that associate with KAR complexes through binding to the GluK1-3 subunits [90, 91]. Neto1 is expressed abundantly in the hippocampus and is a component of postsynaptic KARs at MF-CA3 synapses [91] whereas Neto2 is more highly expressed in the cerebellum [90]. The effects of Neto proteins on KAR channel properties have been studied extensively (reviewed in [92–95]), but, briefly, Neto proteins generally slow the deactivation kinetics of KARs, which accounts for their different properties in vivo compared to exogenously expressed KARs in cell lines that do not contain Netos. Furthermore, it has also recently been shown that Neto proteins control the function of both somatodendritic and presynaptic KARs in somatostatin, cholecystokinin/cannabinoid receptor 1, and parvalbumin-containing interneurons to regulate neuronal network inhibition [96]. However, as outlined below, how Neto proteins affect the trafficking and synaptic incorporation of KARs is less well established with the current literature containing apparently contradictory results.

#### Netos and GluK2

Initial studies observed no effect of Neto1 or Neto2 in mediating surface expression of GluK2 in heterologous systems [90, 91] nor any effects on the abundance of GluK2 or GluK5 in PSD fractions from Neto1 knockout mice [91]. Furthermore, co-expression of Neto1 or Neto2 did not enhance exogenous KAR responses in CA1 pyramidal neurons, which normally lack postsynaptic KAR EPSCs, arguing against a role for Netos in synaptic incorporation of GluK2-containing KARs [9]. Other studies, however, reported a reduction in synaptic GluK2 in the hippocampus of Neto1 knockout mice [97, 98], and from cerebellar PSD fractions from Neto2 knockout mice [99], supporting a role for Neto proteins in the synaptic targeting of GluK2. Moreover, recent studies have reported that Neto1 and Neto2 enhance GluK2 surface expression in HEK293 cells [100] and that injection of Neto2 with GluK2 into oocytes potentiates GluK2 surface expression [77]. Given these apparently contradictory results, the precise roles of Neto proteins in the trafficking and targeting of GluK2-containing KARs remains to be defined.

#### Netos and GluK1

Neto proteins have also been reported to be involved in the trafficking of GluK1-containing KARs. Transfection of Neto2 with GluK1 promotes GluK1 surface expression in both COS-7 cells and cultured hippocampal neurons, and drives the synaptic incorporation of GluK1-containing KARs [101]. Consistent with this role, co-transfection of either Neto1 or Neto2 with GluK1 in CA1 pyramidal cells in hippocampal slice cultures enhances GluK1 surface expression and synaptic targeting [9, 102]. It should be noted, however, that since these studies rely on overexpression of KAR subunits in cells that do not usually express GluK1 [6, 103] or exhibit synaptic KAR responses [6–8], the relevance of Neto proteins to GluK1 trafficking *in vivo* requires further examination.

Overall, although there is not yet a clear consensus, accumulating evidence suggests Neto proteins do influence surface expression and synaptic targeting of KARs under some circumstances. The apparently conflicting data regarding the roles of Netos in KAR trafficking and synaptic positioning can, at least in part, be attributed to the use of different model systems, clonal cell lines and neuronal subtypes, and experimental conditions. Thus, the current inconsistencies may reflect a complex relationship between Netos and KARs, which can be affected differential subunit expression and the availability of cell type-specific interacting proteins. Moreover, GluK2 is subject to multiple, coordinated post-translational modifications (PTMs) including phosphorylation [75], SUMOylation [67, 69], ubiquitination [104, 105] and palmitoylation [71, 106], each of which could potentially directly or indirectly influence the actions of Netos. Clearly, further work is required to will determine the molecular mechanisms, under what circumstances, and for which subunit combinations Netos regulate KAR trafficking and targeting in vivo.

### KAR Post-translational Modifications and Post-synaptic Localisation

#### CaMKII Phosphorylation of GluK5

**Fig. 3:**
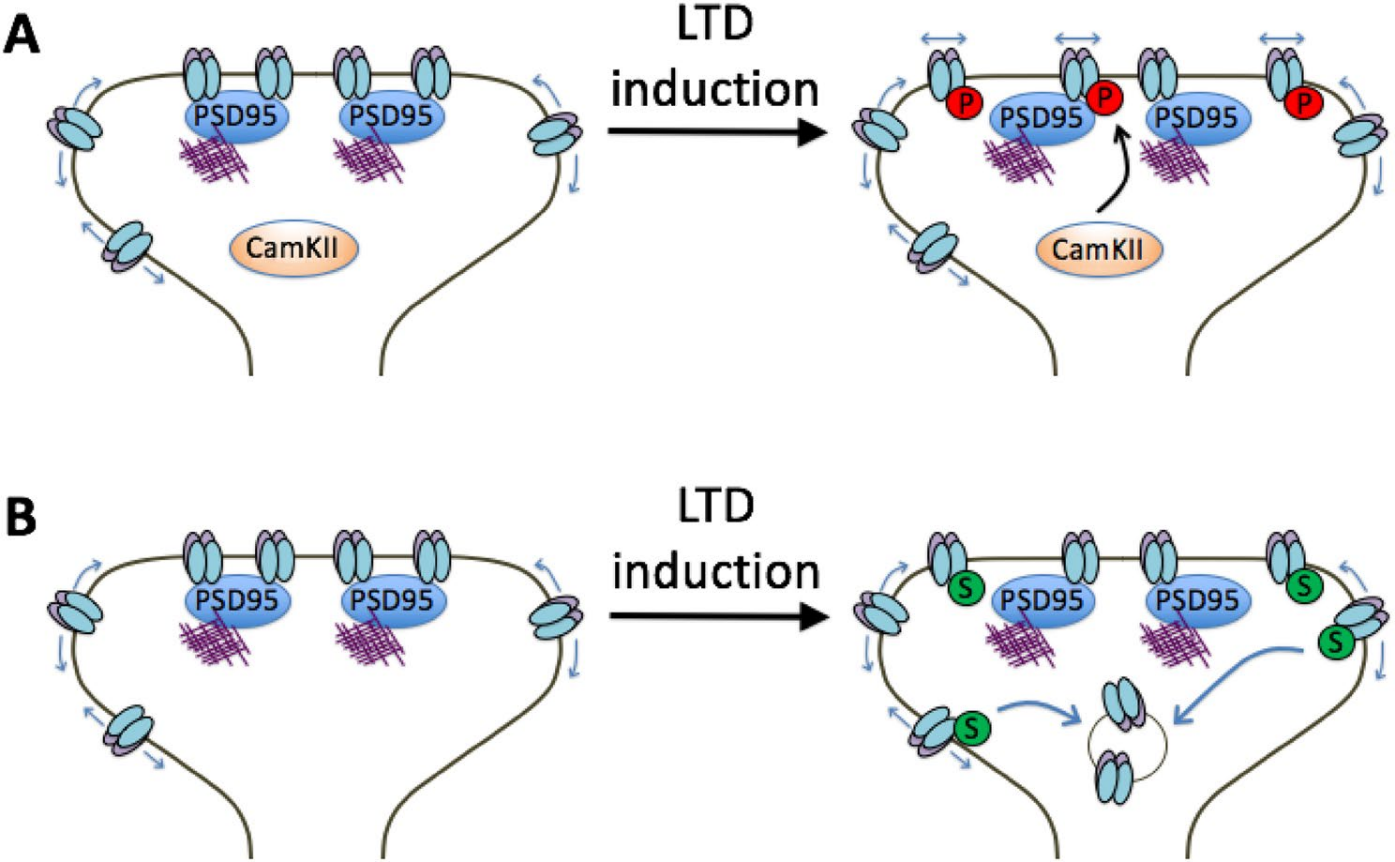
Mechanisms of KAR-LTD. **a** At MF-CA3 synapses, LTD induction leads to CaMKII-mediated phosphorylation of the GluK5 subunit, reducing its ability to bind PSD95. This reduced anchoring of GluK5-containing KARs promotes their lateral mobility and diffusion away from the postsynapse, resulting in LTD of KAR EPSCs. **b** An alternative LTD mechanism results in PKC-dependent phosphorylation (not shown) and subsequent SUMOylation of the GluK2 subunit. This then leads to endocytosis of GluK2-containing KARs and LTD of KAR-mediated synaptic transmission

LTD of KARs at MF-CA3 synapses is induced by a spike timing-, Ca^2+^ influx-, and CaMKII-dependent plasticity mechanism, which is absent in slices from GluK5^−/−^ mice [87]. CaMKII phosphorylates the C-terminal domain of GluK5 in vitro and a phosphomimetic mutation enhances surface expression, but reduces synaptic localisation, in neurons. GluK5 phosphorylation enhances lateral mobility and reduces the interaction between GluK5 and PSD95. Moreover, while re-expression of GluK5 in GluK5^−/−^ slices restored KAR-LTD, expression of a non-phosphorylatable GluK5 did not, demonstrating that direct phosphorylation of GluK5 by CaMKII is required for this form of KAR LTD [87]. Thus, these data indicate that CaMKII phosphorylation of GluK5-containing KARs regulates their synaptic localisation by antagonising the interaction between GluK5 and PSD95 (Fig. 3).

#### PKC Phosphorylation of GluK2

PKC phosphorylation of GluK2 at S846 and S868 regulates the surface expression of GluK2-containing KARs at several levels. It affects both GluK2 transit through the secretory pathway [48, 75] and KAR endocytosis and recycling back to the plasma membrane [15, 75]. Phosphorylation of S846 promotes basal internalisation of GluK2-containing KARs in both HeLa cells and neurons [75], potentially via regulating the interaction between GluK2 and 4.1 proteins [71]. Furthermore, phosphorylation of both sites occurs in response to kainate stimulation of cultured neurons, and phosphorylation at S868 is required for agonist-induced endocytosis of GluK2, by promoting SUMOylation at lysine 886 [15, 69, 107] (see below). It should be noted, however, that PKC phosphorylation of S868 also appears to be involved in recycling of endocytosed GluK2 back to the plasma membrane [15], suggesting that whether phosphorylation of S868 promotes insertion or removal of GluK2 from the plasma membrane is likely to be context-dependent.

#### SUMOylation

SUMOylation is a post-translational modification that results from the conjugation of a member of the ~ 11kD SUMO family to lysine residues in substrate proteins [108]. GluK2 is SUMOylated at a single lysine residue in its intracellular C-terminus, K886, resulting in agonist-induced internalisation of GluK2-containing KARs [67]. Infusion of the catalytic domain of the deSUMOylating enzyme SENP1 increases KAR currents at MF-CA3 synapses, highlighting SUMOylaton as an endogenous regulator of the number of synaptic KARs [67]. Subsequent studies have demonstrated that SUMOylation of GluK2 is enhanced by prior PKC-mediated phosphorylation of serine 868 and is required for LTD of kainate receptors at MF-CA3 synapses [15, 69].

#### Ubiquitination

One major functions of protein ubiquitination is to target proteins for lysosomal or proteasomal degradation [109]. A recent study identified that the Parkinson’s disease-associated ubiquitin ligase Parkin directly interacts with and ubiquitinates the C-terminus of GluK2 [105]. Parkin ubiquitinates GluK2 in both heterologous cells and cultured neurons, and knockdown of Parkin increased GluK2 surface expression and increased vulnerability of hippocampal neurons to kainate-induced excitotoxicity. Furthermore, in a mouse model of autosomal recessive juvenile Parkinson’s expressing a truncated form of Parkin, there are increased levels of GluK2 in substantia nigra and corresponding increases in cortex samples from human patients expressing mutations in Parkin [105]. Thus, GluK2 is a Parkin target that may contribute to the excitotoxic cell death of substantia nigra neurons in Parkinson’s disease.

## KARs in Plasticity

KARs are involved in both excitatory and inhibitory neurotransmission, controlling both short and longer-term plasticity. These properties have been extensively reviewed [2, 11] and we confine our discussion largely to the most recent findings relating to the role of postsynaptic KARs as inducers of long-term plasticity.

### KAR Regulation of Excitatory Neurotransmission

Presynaptic KARs decrease glutamate release at CA3-CA1 pyramidal cell synapses [110–112]. Intriguingly, however, KARs have also been shown to facilitate glutamate release upon application of nanomolar concentrations of kainate. This facilitation of glutamate release requires KAR activation resulting in the accumulation of presynaptic calcium, the production of Ca^2+^–calmodulin complexes and the activation adenylate cyclase and PKA [113–115]. Thus, at certain synapses, KARs can exert bidirectional modulatory actions on glutamate release related to the extent of their activation (for recent review see [3]).

Postsynaptic KARs at MF-CA3 synapses undergo plastic changes, and exhibit several forms of LTD that can be induced by different stimulation protocols [15, 87]. Chamberlain et al. showed that SUMOylation of GluK2 is required for activity-dependent long-term depression of kainate receptor-mediated synaptic transmission (KAR LTD). They We further demonstrated that a critical trigger for SUMOylation is GluK2 phosphorylation by protein kinase C (PKC) and that this sequence of events is required for KAR LTD and that SUMOylation can act as the switch between enhanced or decreased surface expression of KARs after PKC phosphorylation [15].

Furthermore, in cultured neurons, activity-dependent up- or down-regulation of surface KARs can be induced by differential agonist application protocols [13, 68]. KAR surface expression is also subject to homeostatic plasticity and can be ‘scaled’ by manipulating neuronal excitability [48]. Intriguingly, beyond being regulated by plasticity themselves, there is a growing appreciation that KARs also function as postsynaptic inducers of synaptic plasticity (Fig. 3).

Recently, a novel form of AMPAR-LTP was discovered at CA3-CA1 synapses that is mediated by activation of postsynaptic KARs (KAR-LTP_AMPAR_ [16]). Despite the fact that CA1 pyramidal cells exhibit essentially no postsynaptic KAR EPSCs [6, 8, 9, 102, 116], activation of postsynaptic KARs by high-frequency stimulation of Schaffer collaterals inhibits a slow after-hyperpolarization current that regulates excitability in hippocampus (IsAHP) through a metabotropic cascade [117, 118], suggesting receptors are postsynaptically localised. Remarkably, however, at these synapses, KARs, which have a classical ion channel structure, signal primarily through a G protein-dependent pathway (for review see [17]).

**Fig. 4:**
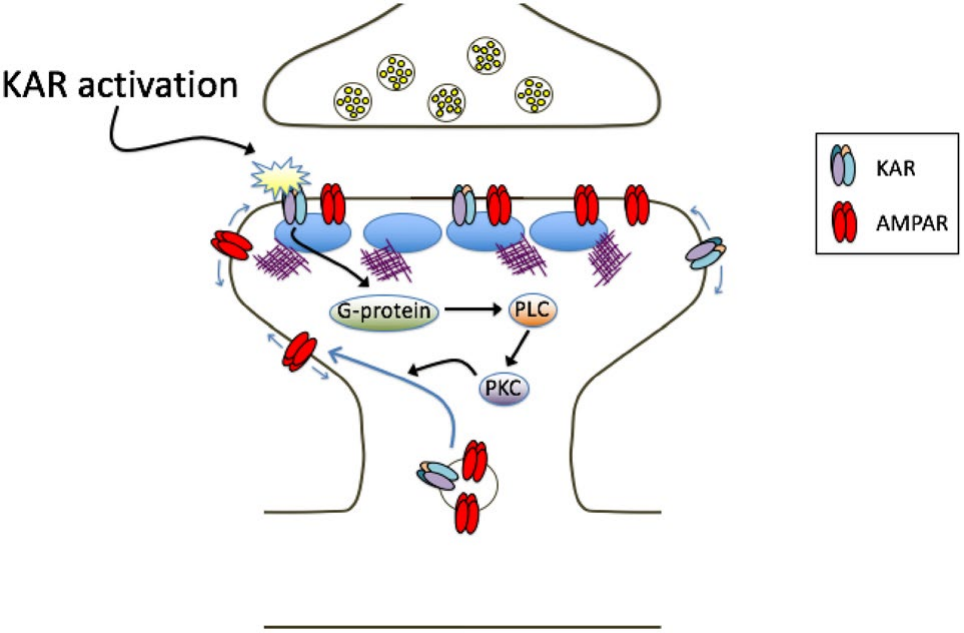
Mechanism of KAR-LTP_AMPAR_. At CA3-CA1 synapses, activation of postsynaptic KARs leads to LTP of AMPAR-mediated synaptic transmission. This occurs through KAR-mediated activation of a pertussis toxin-sensitive G protein, activation of phospholipase C (PLC), PKC, and increased exocytosis of AMPARs from recycling endosomes

Consistent with this, stimulation protocols that activate postsynaptic KARs on CA1 neurons induce LTP of AMPARs via a metabotropic signalling pathway [16]. GluK2^−/−^ mice lack this novel form of plasticity, indicating an absolute requirement for this subunit in induction of KAR-LTP_AMPAR_ (Fig. 4). Similar to inhibition of IsAHP, KAR-LTP_AMPAR_ is mediated by activation of pertussis toxin-sensitive G-proteins, and requires activation of PKC and PLC. Furthermore, similar to LTP induced by activation of NMDARs, KAR-LTP_AMPAR_ is mediated by an increase in surface AMPARs supplied by recruitment of recycling endosomes to spines and also leads to structural plasticity, as determined by an increase in spine size and maturation [16].

Thus, although the exact mechanisms are still to be determined, it is clear that KAR metabotropic signalling plays a key role in directly mediating certain forms of AMPAR-mediated plasticity at CA1 synapses.

### KAR Regulation of Inhibitory Neurotransmission

#### KAR Regulation of GABA Release

An important facet of KAR physiology is that they coordinate and regulate neuronal and network activity via regulation of both excitatory and inhibitory transmission. Presynaptic KARs downregulate GABA release from interneurons in the hippocampus through a metabotropic PKC and PLC dependent pathway that reduces inhibitory postsynaptic currents (IPSCs) [119].

#### KARs and GABA_A_Rs

Moreover, postsynaptic KAR metabotropic signalling depresses synaptic GABA_A_Rs while at the same time potentiating extrasynaptic GABA_A_Rs, in a process that decreases synaptic inhibitory drive to facilitate synaptic plasticity, while simultaneously protecting against neuronal over-excitation by promoting extrasynaptic inhibition [120].

#### KARs and KCC2

In addition to this direct effect on GABA_A_R-mediated transmission, KARs separately regulate the potassium-chloride cotransporter KCC2. KCC2 is crucial because it establishes the electrochemical chloride gradient for postsynaptic inhibition through GABA_A_Rs [121, 122] and its dysregulation is implicated in autism spectrum disorder (ASD; [123]) and epilepsy [124]. KCC2 interacts with both Neto2 [125] and GluK2 [126], which increase the total abundance and enhance surface expression of KCC2 [14]. KCC2 and GluK2 form macromolecular assemblies that traffic together, and this complex regulates intraneuronal chloride homeostasis to support GABA_A_R-mediated transmission [126]. In addition, this process is further controlled by phosphorylation of serines 846 and 868 in GluK2 to determine surface KCC2 levels [14]. These findings establish clear roles for KARs as modulators of postsynaptic inhibitory transmission.

## KARs in Disease

Dysregulation of common molecular pathways underlies multiple neurological and neurodegenerative diseases and there is mounting evidence that KAR dysfunction could be one such feature (reviewed in [127, 128]).

### Epilepsy

KAR dysfunction is particularly closely associated with temporal lobe epilepsy (TLE) [129], and GluK2^−/−^ mice exhibit significantly fewer seizures than wild-type counterparts [79]. TLE is associated with abnormal sprouting of recurrent mossy fibres, which synapse onto dentate gyrus cells [130–132] and this abnormal sprouting is also reduced in GluK2^−/−^ mice [133]. Consistent with the requirement for recruitment of KARs to postsynaptic sites for seizure activity, despite induction of MF sprouting in an animal model of TLE, C1ql2/3 double KO mice are resistant to seizures [89] and genetic silencing of KARs at CA3 synapses attenuates kainate-induced seizures [134]. Together, these studies suggest that recurrent synaptic activity, mediated by postsynaptic KARs, drives seizure activity in TLE, and highlights KARs as an attractive therapeutic target in the treatment of this disorder.

### KARs and Other Neurological Disorders

In addition to TLE, both gain [135] and loss [136, 137] of GluK2 have been reported to lead to ASD-like phenotypes. Furthermore, duplication of the GluK4 gene has been associated with autism [138] and, consistent with this, overexpression of GluK4 in the forebrain of mice causes severe anxiety and ASD-like behaviours [139]. Additionally, GluK4 has been linked with treatment-resistant depression [140], and disruption of the gene encoding GluK4 has been observed in a patient with schizophrenia and mental retardation [141].

Most recently it has been reported that complete ablation of all KAR subunits results in disrupted corticostriatal function and dramatic obsessive-compulsive-like behaviour with severe self-grooming [142]. Together with the previous studies, these new observations support the notion that KAR-mediated control of network activity is a key determinant of higher brain function, and that disruption of these pathways can lead to multiple neurological disorders.

## Perspectives

Although historically much less studied than AMPARs or NMDARs, there is a currently a renaissance in KAR research fuelled by the realisation that they are multifunctional neuronal modulators that have roles in health and disease far beyond those previously appreciated.

To understand how KARs exert these regulatory effects, it is necessary to define how KARs themselves are controlled and how they interact with, and regulate, other proteins and systems. Furthermore, while there is a growing appreciation of the multifaceted roles played by KARs, a number of fundamental questions remain unanswered. For example, while it is widely-accepted that KARs can signal both ionotropically and metabotropically through G-proteins, how this dual signaling occurs, and what determines which ‘mode’ of signaling an individual KAR complex utilizes remains almost entirely unexplored. In addition, while we are beginning to understand the molecular events that control the specific distribution and synaptic localization of KARs, how these mechanisms cooperate to orchestrate the highly selective distribution of KARs in the brain remains an important unanswered question. Finally, although KARs can induce plasticity of AMPARs, exactly how this occurs, how widespread this form of plasticity is in the brain, and whether postsynaptic KARs can direct other forms of bidirectional synaptic plasticity remain unknown.

We expect that further studies examining how KAR localisation and signalling at pre- and postsynaptic sites impacts on neuronal function will answer many of these remaining questions, and will transform our understanding of the roles KARs play in development, plasticity and disease.
